# (*E*)-1-(3-Cyano­benzyl­idene)thio­semi­carbazide *N*,*N*-dimethyl­formamide solvate

**DOI:** 10.1107/S160053681000214X

**Published:** 2010-01-23

**Authors:** Mei Shi

**Affiliations:** a Department of Chemistry, Nanjing Xiaozhuang University, Nanjing 210017, People’s Republic of China

## Abstract

The title compound, C_9_H_8_N_4_S·C_3_H_7_NO, adopts an *E* configuration about both the C=N and C—N bonds. Inter­molecular N—H⋯O hydrogen bonding links the compound to the DMF solvent molecule. The crystal packing is characterized by chains of mol­ecules linked by inter­molecular N—H⋯S hydrogen-bonding inter­actions.

## Related literature

For the biological activity of thio­semicarbazones, see: Lovejoy & Richardson *et al.* (2002 ▶). For a related structure, see: Wu *et al.* (2009 ▶). For comparitive geometrical parameters, see: Sutton *et al.* (1965 ▶).
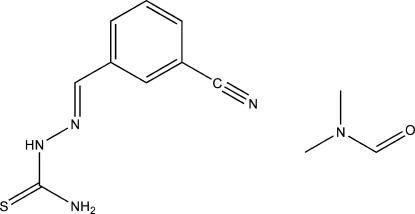


## Experimental

### 

#### Crystal data

C_9_H_8_N_4_S·C_3_H_7_NO
*M* *_r_* = 277.35
Monoclinic, 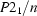

*a* = 7.312 (7) Å
*b* = 8.945 (3) Å
*c* = 22.316 (19) Å
β = 92.12 (2)°
*V* = 1458.6 (19) Å^3^
*Z* = 4
Mo *K*α radiation
μ = 0.22 mm^−1^
*T* = 293 K
0.20 × 0.20 × 0.20 mm

#### Data collection

Rigaku Mercury2 diffractometer
Absorption correction: multi-scan (*CrystalClear*; Rigaku, 2005 ▶) *T*_min_ = 0.742, *T*_max_ = 1.000
9561 measured reflections
3280 independent reflections
2065 reflections with *I* > 2σ(*I*)
*R*_int_ = 0.052

#### Refinement

*R*[*F* ^2^ > 2σ(*F* ^2^)] = 0.055
*wR*(*F* ^2^) = 0.109
*S* = 1.01
3280 reflections
172 parameters
H-atom parameters constrained
Δρ_max_ = 0.18 e Å^−3^
Δρ_min_ = −0.20 e Å^−3^

### 

Data collection: *CrystalClear* (Rigaku, 2005 ▶); cell refinement: *CrystalClear*; data reduction: *CrystalClear*; program(s) used to solve structure: *SHELXS97* (Sheldrick, 2008 ▶); program(s) used to refine structure: *SHELXL97* (Sheldrick, 2008 ▶); molecular graphics: *SHELXTL* (Sheldrick, 2008 ▶); software used to prepare material for publication: *SHELXTL*.

## Supplementary Material

Crystal structure: contains datablocks I, global. DOI: 10.1107/S160053681000214X/pv2253sup1.cif

Structure factors: contains datablocks I. DOI: 10.1107/S160053681000214X/pv2253Isup2.hkl

Additional supplementary materials:  crystallographic information; 3D view; checkCIF report

## Figures and Tables

**Table 1:** Hydrogen-bond geometry (Å, °)

*D*—H⋯*A*	*D*—H	H⋯*A*	*D*⋯*A*	*D*—H⋯*A*
N3—H3*A*⋯O1	0.86	1.96	2.795 (3)	162
N4—H4*A*⋯N1^i^	0.86	2.35	3.101 (3)	146
N4—H4*B*⋯S1^ii^	0.86	2.59	3.364 (2)	150
C8—H8*A*⋯O1	0.93	2.54	3.293 (3)	138

Symmetry codes: (i) 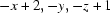; (ii) 
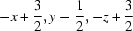
.

**Fig. 1.**
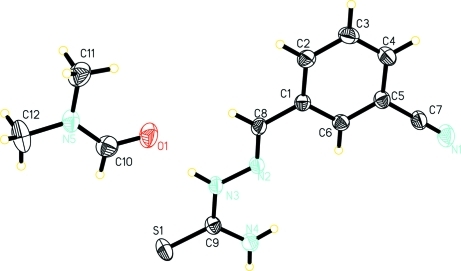
Perspective structure of the title compound, showing the atom-numbering scheme. Displacement ellipsoids are drawn at the 30% probability level.

**Fig. 2.**
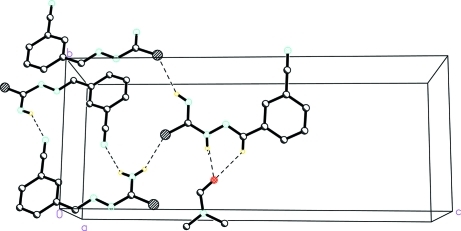
The crystal packing of the title compound viewed along the a axis showing the two-dimensionnal hydrogen bondings network. H-atoms non involved in H-bonding interactions have been excluded for clarity.

## supplementary crystallographic information

### Comment

The antiproliferative activity of a series of thiosemicarbazones has been reported (Lovejoy & Richardson, 2002). As a research on thiosemicarbazones, the synthesis and crystal structure of a new Schiff base compound derived from thiosemicarbazide and 3-cyanobenzaldehyde has been presented in this article. The crystal structure of 4-cyanobenzaldehyde thiosemicarbazone which is closely related to the title compound has been reported recently (Wu *et al.* 2009).

The thiosemicarbazone moiety in the title compound (Fig. 1) is nearly planar and shows an E configuration about both the C9—N3 and C8═N2 bonds. The C—S bond distance of 1.680 (2) Å agrees well with similar bonds in related compounds, being intermediate between 1.82 Å for a C—S single bond and 1.56 Å for a C═S double bond (Sutton *et al.* 1965). All the bond distances except for the C6—C9 (bond length, 1.448 (3) Å) fall within the normal range. The intermolecular N—H···O hydrogen bond stabilizes the molecular conformation. In the crystal packing, adjacent molecules are linked by N—H···S hydrogen bonds (Table 1 and Fig. 2) to form chains running parallel to the *a* axis. Weak interactions of the type C—H···O are also present in the structure.

### Experimental

The title compound was synthesized by refluxing 3-cyanobenzaldehyde (2.1 g, 16 mmol) and thiosemicarbazide (1.46 g, 16 mmol) in absolute ethanol (50 ml) for 10 h. After cooling to room temperature, the white solid formed was isolated and dried under vacuum. The title compound was isolated using column chromatography (petroleum ether: ethyl acetate-2:1). Single crystals suitable for X-ray diffraction analysis were obtained from slow evaporation of DMF solution.

### Refinement

H atoms were placed in calculated positions and refined using a riding model, with N—H = 0.86 Å, C—H = 0.93–0.96 Å and with *U*_iso_(H) = 1.2 and 1.5 times *U*_eq_ of nonmethyl and methyl type H-atoms.

### Crystal data

**Table:**

C_9_H_8_N_4_S·C_3_H_7_NO	*F*(000) = 584
*M**_r_* = 277.35	*D*_x_ = 1.263 Mg m^−^^3^
Monoclinic, *P*2_1_/*n*	Mo *K*α radiation, λ = 0.71073 Å
Hall symbol: -P 2yn	Cell parameters from 2851 reflections
*a* = 7.312 (7) Å	θ = 2.3–27.4°
*b* = 8.945 (3) Å	µ = 0.22 mm^−^^1^
*c* = 22.316 (19) Å	*T* = 293 K
β = 92.12 (2)°	Block, pale yellow
*V* = 1458.6 (19) Å^3^	0.20 × 0.20 × 0.20 mm
*Z* = 4	

### Data collection

**Table:**

Rigaku Mercury2 diffractometer	3280 independent reflections
Radiation source: fine-focus sealed tube	2065 reflections with *I* > 2σ(*I*)
graphite	*R*_int_ = 0.052
Detector resolution: 13.6612 pixels mm^-1^	θ_max_ = 27.4°, θ_min_ = 2.5°
CCD_Profile_fitting scans	*h* = −7→9
Absorption correction: multi-scan (*CrystalClear*; Rigaku, 2005)	*k* = −11→11
*T*_min_ = 0.742, *T*_max_ = 1.000	*l* = −28→24
9561 measured reflections	

### Refinement

**Table:**

Refinement on *F*^2^	Primary atom site location: structure-invariant direct methods
Least-squares matrix: full	Secondary atom site location: difference Fourier map
*R*[*F*^2^ > 2σ(*F*^2^)] = 0.055	Hydrogen site location: inferred from neighbouring sites
*wR*(*F*^2^) = 0.109	H-atom parameters constrained
*S* = 1.01	*w* = 1/[σ^2^(*F*_o_^2^) + (0.020*P*)^2^ + 0.850*P*] where *P* = (*F*_o_^2^ + 2*F*_c_^2^)/3
3280 reflections	(Δ/σ)_max_ = 0.001
172 parameters	Δρ_max_ = 0.18 e Å^−^^3^
0 restraints	Δρ_min_ = −0.20 e Å^−^^3^

### Special details

**Table:**

Geometry. All e.s.d.'s (except the e.s.d. in the dihedral angle between two l.s. planes) are estimated using the full covariance matrix. The cell e.s.d.'s are taken into account individually in the estimation of e.s.d.'s in distances, angles and torsion angles; correlations between e.s.d.'s in cell parameters are only used when they are defined by crystal symmetry. An approximate (isotropic) treatment of cell e.s.d.'s is used for estimating e.s.d.'s involving l.s. planes.
Refinement. Refinement of *F*^2^ against ALL reflections. The weighted *R*-factor *wR* and goodness of fit *S* are based on *F*^2^, conventional *R*-factors *R* are based on *F*, with *F* set to zero for negative *F*^2^. The threshold expression of *F*^2^ > σ(*F*^2^) is used only for calculating *R*-factors(gt) *etc*. and is not relevant to the choice of reflections for refinement. *R*-factors based on *F*^2^ are statistically about twice as large as those based on *F*, and *R*- factors based on ALL data will be even larger.

### Fractional atomic coordinates and isotropic or equivalent isotropic displacement parameters (Å^2^)

**Table:**

	*x*	*y*	*z*	*U*_iso_*/*U*_eq_	
S1	0.60691 (11)	0.46135 (8)	0.74146 (3)	0.0636 (2)	
N2	0.7389 (2)	0.40996 (19)	0.57477 (8)	0.0425 (4)	
N3	0.6727 (2)	0.4707 (2)	0.62652 (8)	0.0451 (5)	
H3A	0.6198	0.5566	0.6258	0.054*	
C6	0.8765 (3)	0.2925 (2)	0.46844 (10)	0.0430 (5)	
H6A	0.8857	0.2327	0.5025	0.052*	
N4	0.7782 (3)	0.2634 (2)	0.67556 (9)	0.0585 (6)	
H4A	0.8181	0.2320	0.6420	0.070*	
H4B	0.7939	0.2102	0.7074	0.070*	
C8	0.7256 (3)	0.4903 (2)	0.52748 (10)	0.0428 (5)	
H8A	0.6724	0.5846	0.5288	0.051*	
C9	0.6924 (3)	0.3936 (2)	0.67810 (10)	0.0441 (5)	
C7	1.0229 (3)	0.0938 (3)	0.41270 (10)	0.0518 (6)	
C1	0.7947 (3)	0.4329 (2)	0.47102 (9)	0.0402 (5)	
N1	1.0774 (3)	−0.0249 (3)	0.41178 (10)	0.0714 (7)	
C5	0.9444 (3)	0.2423 (2)	0.41459 (10)	0.0444 (5)	
C2	0.7832 (3)	0.5198 (3)	0.41938 (10)	0.0501 (6)	
H2B	0.7295	0.6140	0.4207	0.060*	
C3	0.8504 (3)	0.4685 (3)	0.36610 (10)	0.0565 (6)	
H3B	0.8407	0.5281	0.3320	0.068*	
C4	0.9316 (3)	0.3298 (3)	0.36311 (10)	0.0535 (6)	
H4C	0.9770	0.2953	0.3273	0.064*	
N5	0.4289 (3)	0.9814 (2)	0.63750 (9)	0.0565 (5)	
C10	0.4945 (4)	0.8452 (3)	0.64567 (13)	0.0641 (7)	
H10A	0.4904	0.8055	0.6841	0.077*	
O1	0.5606 (3)	0.76602 (19)	0.60695 (9)	0.0718 (6)	
C11	0.4348 (4)	1.0502 (3)	0.57878 (13)	0.0756 (8)	
H11A	0.4869	0.9813	0.5512	0.113*	
H11B	0.5085	1.1389	0.5813	0.113*	
H11C	0.3129	1.0759	0.5650	0.113*	
C12	0.3501 (4)	1.0680 (4)	0.68517 (15)	0.0925 (11)	
H12A	0.3521	1.0099	0.7214	0.139*	
H12B	0.2261	1.0936	0.6740	0.139*	
H12C	0.4202	1.1577	0.6917	0.139*	

### Atomic displacement parameters (Å^2^)

**Table:**

	*U*^11^	*U*^22^	*U*^33^	*U*^12^	*U*^13^	*U*^23^
S1	0.0917 (5)	0.0516 (4)	0.0489 (4)	−0.0029 (4)	0.0207 (3)	−0.0114 (3)
N2	0.0512 (12)	0.0372 (9)	0.0396 (10)	0.0037 (8)	0.0069 (9)	−0.0025 (8)
N3	0.0550 (12)	0.0366 (9)	0.0444 (11)	0.0082 (9)	0.0102 (9)	−0.0053 (8)
C6	0.0502 (14)	0.0383 (12)	0.0403 (12)	0.0002 (10)	0.0011 (10)	0.0022 (9)
N4	0.0848 (16)	0.0461 (11)	0.0454 (12)	0.0152 (11)	0.0121 (11)	0.0056 (9)
C8	0.0450 (13)	0.0359 (12)	0.0477 (14)	0.0064 (10)	0.0030 (10)	−0.0018 (10)
C9	0.0508 (15)	0.0351 (11)	0.0465 (13)	−0.0047 (10)	0.0054 (11)	−0.0038 (10)
C7	0.0604 (16)	0.0490 (14)	0.0468 (14)	0.0050 (12)	0.0114 (12)	−0.0048 (11)
C1	0.0421 (13)	0.0373 (12)	0.0413 (12)	0.0011 (9)	0.0014 (10)	−0.0015 (9)
N1	0.0898 (18)	0.0539 (14)	0.0718 (16)	0.0190 (13)	0.0214 (13)	−0.0032 (12)
C5	0.0486 (14)	0.0392 (12)	0.0454 (13)	0.0005 (10)	0.0031 (11)	−0.0050 (10)
C2	0.0603 (16)	0.0407 (12)	0.0492 (14)	0.0063 (11)	−0.0003 (12)	0.0025 (11)
C3	0.0730 (18)	0.0554 (15)	0.0408 (14)	0.0039 (13)	0.0006 (12)	0.0081 (12)
C4	0.0642 (17)	0.0558 (15)	0.0411 (14)	−0.0010 (13)	0.0075 (12)	−0.0042 (11)
N5	0.0650 (14)	0.0431 (11)	0.0621 (14)	0.0022 (10)	0.0117 (11)	−0.0079 (10)
C10	0.074 (2)	0.0515 (16)	0.0665 (18)	−0.0059 (14)	0.0011 (15)	0.0052 (13)
O1	0.0870 (15)	0.0432 (10)	0.0860 (14)	0.0116 (10)	0.0131 (11)	−0.0066 (10)
C11	0.088 (2)	0.0541 (17)	0.086 (2)	0.0066 (15)	0.0132 (17)	0.0126 (15)
C12	0.095 (2)	0.083 (2)	0.102 (3)	−0.0076 (19)	0.034 (2)	−0.0443 (19)

### Geometric parameters (Å, °)

**Table:**

S1—C9	1.680 (2)	C2—C3	1.382 (3)
N2—C8	1.277 (3)	C2—H2B	0.9300
N2—N3	1.380 (2)	C3—C4	1.378 (3)
N3—C9	1.345 (3)	C3—H3B	0.9300
N3—H3A	0.8600	C4—H4C	0.9300
C6—C5	1.392 (3)	N5—C10	1.320 (3)
C6—C1	1.393 (3)	N5—C11	1.450 (3)
C6—H6A	0.9300	N5—C12	1.452 (3)
N4—C9	1.325 (3)	C10—O1	1.230 (3)
N4—H4A	0.8600	C10—H10A	0.9300
N4—H4B	0.8600	C11—H11A	0.9600
C8—C1	1.467 (3)	C11—H11B	0.9600
C8—H8A	0.9300	C11—H11C	0.9600
C7—N1	1.135 (3)	C12—H12A	0.9600
C7—C5	1.447 (3)	C12—H12B	0.9600
C1—C2	1.390 (3)	C12—H12C	0.9600
C5—C4	1.390 (3)		
			
C8—N2—N3	116.85 (18)	C1—C2—H2B	119.5
C9—N3—N2	118.97 (18)	C4—C3—C2	120.5 (2)
C9—N3—H3A	120.5	C4—C3—H3B	119.7
N2—N3—H3A	120.5	C2—C3—H3B	119.7
C5—C6—C1	119.6 (2)	C3—C4—C5	118.9 (2)
C5—C6—H6A	120.2	C3—C4—H4C	120.6
C1—C6—H6A	120.2	C5—C4—H4C	120.6
C9—N4—H4A	120.0	C10—N5—C11	119.6 (2)
C9—N4—H4B	120.0	C10—N5—C12	122.9 (3)
H4A—N4—H4B	120.0	C11—N5—C12	117.5 (2)
N2—C8—C1	119.7 (2)	O1—C10—N5	125.8 (3)
N2—C8—H8A	120.2	O1—C10—H10A	117.1
C1—C8—H8A	120.2	N5—C10—H10A	117.1
N4—C9—N3	116.8 (2)	N5—C11—H11A	109.5
N4—C9—S1	123.00 (18)	N5—C11—H11B	109.5
N3—C9—S1	120.24 (17)	H11A—C11—H11B	109.5
N1—C7—C5	177.1 (3)	N5—C11—H11C	109.5
C2—C1—C6	118.9 (2)	H11A—C11—H11C	109.5
C2—C1—C8	120.3 (2)	H11B—C11—H11C	109.5
C6—C1—C8	120.8 (2)	N5—C12—H12A	109.5
C4—C5—C6	121.1 (2)	N5—C12—H12B	109.5
C4—C5—C7	120.5 (2)	H12A—C12—H12B	109.5
C6—C5—C7	118.4 (2)	N5—C12—H12C	109.5
C3—C2—C1	121.0 (2)	H12A—C12—H12C	109.5
C3—C2—H2B	119.5	H12B—C12—H12C	109.5

### Hydrogen-bond geometry (Å, °)

**Table:**

*D*—H···*A*	*D*—H	H···*A*	*D*···*A*	*D*—H···*A*
N3—H3A···O1	0.86	1.96	2.795 (3)	162
N4—H4A···N2	0.86	2.25	2.610 (3)	105
N4—H4A···N1^i^	0.86	2.35	3.101 (3)	146
N4—H4B···S1^ii^	0.86	2.59	3.364 (2)	150
C8—H8A···O1	0.93	2.54	3.293 (3)	138
C11—H11A···O1	0.96	2.34	2.767 (3)	106

Symmetry codes: (i) −*x*+2, −*y*, −*z*+1; (ii) −*x*+3/2, *y*−1/2, −*z*+3/2.
